# Integrating palliative care into primary care for older people with multimorbid serious illness: a multinational qualitative cross-sectional study in Sub-Saharan Africa

**DOI:** 10.1136/bmjph-2024-001355

**Published:** 2025-03-23

**Authors:** Kennedy Bashan Nkhoma, Maya Jane Bates, Dorothee van Breevoort, Dickson Dick Chifamba, Catherine J Evans, Duncan Kwaitana, Adwoa Bemah Boamah Mensah, Modai Clement Mnenula, Lovemore Mupaza, Edwina Beryl Addo Opare-Lokko, Richard Harding

**Affiliations:** 1 Florence Nightingale Faculty of Nursing, Midwifery and Palliative Care, Cicely Saunders Institute, King’s College London, London, London, UK; 2 Family Medicine, Kamuzu University of Health Sciences, Blantyre, Southern Region, Malawi; 3 Clinical Sciences, Liverpool School of Tropical Medicine, Liverpool, UK; 4 Island Hospice and Healthcare, Harare, Zimbabwe; 5 Sussex Community NHS Foundation Trust, Brighton, Brighton and Hove, UK; 6 Nursing, Kwame Nkrumah University of Science and Technology, Kumasi, Ashanti, Ghana; 7 Ghana College of Physicians and Surgeons, Accra, Ghana

**Keywords:** Cross-Sectional Studies, Age Factors, Comorbidity, Communication

## Abstract

**Background:**

The WHO primary palliative care strategy states that palliative care is ‘an ethical responsibility of health systems’ and calls for integration of palliative care into public healthcare systems to achieve universal health coverage. We aimed to determine stakeholders’ perspectives on the necessary components of and considerations for a feasible and acceptable model of integrated palliative care and primary care for older people living with serious multimorbid illness in Sub-Saharan Africa.

**Methods:**

We conducted a multicountry cross-sectional qualitative study in Ghana, Malawi and Zimbabwe. In-depth qualitative interviews were conducted with multimorbid older people and family caregivers. Focus groups were conducted with healthcare staff. Verbatim transcripts were subjected to inductive framework analysis to identify stakeholders’ needs and preferences for delivering and receiving palliative care in primary care facilities.

**Results:**

The coding framework identified five main themes: (i) communication; (ii) coordination of care; (iii) impact of living with chronic illness; (iv) seeking healthcare; and (v) living with chronic illness: coping strategies and resources. The impact of multimorbid illness on older people was multidimensional, including pain and symptom control, catastrophic spending, social exclusion and limitations on activities of daily living. Specific challenges were identified in care pathways and delivery. Communication was sub-optimal, with lack of appropriate information and patient involvement.

**Conclusion:**

Person-centred approaches are required to deliver palliative care to older multimorbid people in primary care settings. This study informs implementation of the WHO Healthy Ageing Policy intention to deliver person-centred primary palliative care and the WHO primary palliative care guidance.

**WHAT IS ALREADY KNOWN ON THIS TOPIC**

- Successes to date in global health bring a new challenge, that is, how to meet the needs of ageing patients with increasingly complex multimorbidity. The prevalence and burden of complex serious multimorbid conditions increase with age. These patients and their families face a high burden of physical symptoms and psychosocial problems and high formal and informal health service use.
- Only 10% of the 20 million people each year who require palliative care receive it, and while most provision is in high-income countries, 80% of need is in low- and middle-income countries.
- Strengthening primary health care is vital to improve outcomes and reduce inequity, especially for remote communities. Primary care utilisation for chronic disease management can improve patient outcomes and reduce costs, providing holistic person-centred care to reduce secondary and tertiary care use.

**WHAT THIS STUDY ADDS**

- The impact of multimorbid illness on older people was multidimensional, including pain and symptom control, catastrophic spending, social exclusion and limitations on activities of daily living. Specific challenges were identified in care pathways and delivery.
- Communication was sub-optimal, with lack of appropriate information and patient involvement in decision-making about their care. Primary care clinics required older people to physically move through many steps of the primary care pathway with good mobility and physical capacity to attend for long periods.

**HOW MIGHT THIS STUDY AFFECT RESEARCH, PRACTICE OR POLICY**

- Person-centred approaches are required to deliver palliative care to older multimorbid people in primary care settings. Research is needed to deliver a feasible and appropriate model of care that integrates palliative care suitable for older people in primary care settings.
- The multidimensional nature of patient needs requires detailed person-centred assessment to care. Care delivery requires staff to utilise person-centred communication skills that empower patients and families, within care pathways that are adapted to the needs of older people.

## Background

 The WHO estimates that by 2050, 80% of older people will be living in low- and middle-income countries (LMICs).^1^ Analysis of WHO mortality data has projected that from 2016 to 2060, the number of people dying with serious health-related suffering will rise from over 20 million to 39 million deaths in LMICs (83% of all global deaths).^2^ The greatest rise (ie, 153%) is projected in low-income countries. Serious physical illness will account for seven out of the 10 most common conditions driving the increase in serious health-related suffering in LMICs. These increases will be largely in progressive and life-limiting diseases of ageing with high symptom prevalence and burden (eg, cancer, cerebrovascular and lung disease and dementia).^3^

Palliative care is recognised as an essential health service within universal health coverage (UHC).^4^ UHC is the third goal of the sustainable development goals and aims to ensure healthy lives and promote well-being for children and adults including older people.^5^ Older people typically experience rising multimorbidity as they age, leading to high usage of informal and formal health services.^6^

The Academy of Medical Sciences (AMS) states that palliative care pathways (which commonly provide multimorbid person-centred care) may offer important models to meet increasing population-level needs.^7^ A systematic review recommended research to determine how findings of benefit for early integrated palliative care can be best translated into feasible models in LMICs.^8^ Although palliative care is a critical component of global health and public health systems,^9^ evidence to date in Sub-Saharan Africa has focused largely on management of people with HIV disease.^10^ There is little evidence for older people and/or multimorbidity in the region.^11^

Palliative care is less accessible, under-utilised and unintegrated in many of the healthcare systems in Sub-Saharan Africa. A recent review of barriers to palliative care services in Africa found three levels of barriers: (1) individual-level, (2) system-level and (3) relational barriers. The studies reviewed predominantly used quantitative cross-sectional and retrospective study design, hence the need for new evidence employing qualitative design.^12^ Another review classified barriers into seven categories which included funding and physical resources, human resources and training, cultural norms, statutory barriers, processes and networks, and community.^13^ A previous review of palliative care in Africa^14^ to assess the progress of palliative care between 2005 and 2016 reported data on medication availability, education, policies, vitality, service provision and implementation. It concluded that palliative care provision is affected by restricted access to opioids such as morphine and prescriber restriction laws. They further concluded that information on palliative care services is unevenly distributed; however, the available information shows that palliative care services have increased in a subset of African countries including Malawi, Mozambique, Rwanda, Swaziland, Tanzania, Zimbabwe, Uganda, Tanzania, Kenya and South Africa. There is still minimal or no identified palliative care development in most African countries.^14^

A recent review of the state of palliative care in Malawi showed that Malawi has implemented diverse strategies across all pillars to develop palliative care including creating a national stand-alone palliative care policy; integrating palliative care into the curricula of nursing and medical professionals; developing and implementing training for diverse service providers; establishing systems for the procurement and distribution of opioids; training on opioids prescription; and implementing diverse models of palliative care service delivery.^15^ However, there is a lack of local evidence to inform palliative care, for example, palliative care integration for older people in primary care settings.^13^ Similar findings have been reported in East Africa.^16^

The Alma Ata conference declared primary healthcare as the central function and focus of health systems.^17^ Health systems strengthening through primary care in LMICs is important to improve outcomes for older people as they may use these services frequently.^18 19^ Primary care utilisation for chronic disease management can improve patient outcomes and reduce costs.^20^ The WHO has identified primary care as the route to stronger health systems and through which palliative care can be best delivered.^5 21^

The concepts of primary healthcare and palliative care have similar principles: primary healthcare promotes self-determination, patient engagement, and participation in care and recognises the interrelationship between physical, social and economic development.^22^

We have summarised key concepts in table 1 to contextualise the concept of primary palliative care in Sub-Saharan Africa.^22 23^

**Table 1:** Primary palliative care in the African context: adapted from^23 27^

Concept	Description
Primary palliative care	Primary palliative care is palliative care practised by primary healthcare workers, who are the principal providers of integrated healthcare for people in local communities throughout their life. The services include early identification and triggering of palliative care as part of integrated and holistic chronic disease management, referral and collaborating with specialist palliative care services where they exist, and strengthening underlying professional capabilities in primary care.^22^Care consists of management of physical, psychological, social, and spiritual problems and concerns among patients with life-threatening illnesses. Care is provided in all settings: hospitals, health centres, clinics and at home, thus preventing unnecessary hospital admissions. Community involvement is key such as volunteers and community health workers.
Generalist/basic palliative care	Care provided by healthcare professionals specifically trained to manage patients with life-threatening diseases and more complex palliative care needs. They train other staff to provide palliative care and act as reference points for patients and other hospital services.They mainly work in primary care settings, typically district hospitals which serve as referral settings for health centres.They also provide services to patients and families in their communities or homes. In African primary care settings, these services are provided by a clinical officer/doctor, nurse and volunteers.^27^
Specialised palliative care	Care is provided by a specialist multidisciplinary and interdisciplinary team to patients with complex palliative care needs. These teams are either basic (doctor and nurse) or more complex, having other components (psychologists, social workers, spiritual counsellors, occupational therapists, physiotherapists, pharmacists and others) with various possible degrees of involvement. These services are not available in primary care settings such as district hospitals.^27^
Integrated palliative care	Provision of palliative care into all services and settings and with coordination of care. Integrated palliative care aims to join up the health and care services required by individuals to deliver care that meets their personal needs in an efficient way.^23^

The healthcare delivery system is similar across the three countries; they all have three main levels of care: primary (clinics, health centres, health post), secondary (district hospitals, community hospitals, regional hospitals) and tertiary (teaching hospitals, specialist hospitals).

All levels are either owned by the government, church institutions or private sectors. The majority in each country are owned by the government. In Ghana across the country, 54% are government facilities, 40% are privately owned, while faith-based account for 10%.^24^ In Malawi, 68% of the facilities are owned by the government, while 32% are faith-based facilities, and 10% are privately owned.^25^ In Zimbabwe, 56% are government owned, 35% are faith-based, while 9% are privately owned. The governments have a national healthcare service which is government funded and free to all citizens at the point of delivery.^26^

This paper presents the development phase that aimed to determine primary care staff, patients’ and families’ perspectives on the necessary components of and considerations for a feasible and acceptable model of integrated primary palliative care for older people living with serious multimorbid illness in Sub-Saharan Africa.

For this work, we aimed to integrate palliative care at service, clinical and horizontal levels as described in table 2.^27 28^

**Table 2:** Types and levels of palliative care integration^27^

Types and levels of integration	Description
Service integration	Involves the coordination of different services, such as through multidisciplinary teams, single referral structures or single clinical assessment processes.
Clinical integration	Involves the coordination of care into a single or coherent process, either within or across professions. This could involve developing shared guidelines or protocols across boundaries of care.
Horizontal integration	Integration between organisations, networks or groups within the health sector, usually at the same level of care. For this work, the focus was between a district hospital, health centre and home-based care

## Methods

### Study design

This cross-national, cross-sectional study using qualitative in-depth face-to-face semistructured interviews and focus group discussions and framework analysis was informed by the Medical Research Council guidance on complex intervention development.^29^ It is reported in line with the COnsolidated criteria for Reporting Qualitative research and Standards for Reporting Qualitative Research checklists.^30^

### Setting

Primary care clinics within hospitals in Ghana, Malawi and Zimbabwe. Across all settings, primary care is the first point of contact with a healthcare provider. All these facilities are district hospitals which also serve as referral sites for health centres. Doctors and registered nurses manage the district hospitals.

### Study participants

We recruited from three populations: (1) older people with serious illness, (2) family caregivers of older people with serious illness and (3) healthcare professionals working with older people in primary care.

#### Older people with serious illness

We define serious illness as ‘a condition that carries a high risk of mortality, negatively impacts quality of life and daily function, and/or is burdensome in symptoms, treatments or caregiver stress’.^31^

Inclusion criteria: aged 50 years and above, in line with the WHO definition of older people for research in Africa.^32^

Living with two or more serious illnesses/chronic illnesses, in line with the AMS definition of multimorbidity, that is, *‘*a scenario whereby a person experiences any possible combination of chronic conditions, which could encompass diagnosed and undiagnosed physical, infectious, and mental health conditions. Within any one person, these component conditions may or may not interact with each other, either in their pathophysiology, clinical management, or impact on the patient*’*.^33^

Exclusion criteria: those below 50 years of age and living with only one or no serious illness as defined by the AMS, too unwell or distressed to participate, or provide informed consent.

#### Family caregivers of older people with serious illness

We define family caregivers as ‘unpaid, informal providers of one or more physical, social, practical and emotional tasks. In terms of their relationship to the patient, they may be a friend, partner, ex-partner, sibling, parent, child or other blood or non-blood relative’.^34^

Other eligibility criteria for family caregivers (hereafter referred to as caregivers) included at least 18 years of age and responsible for day-to-day informal care provision to older people. Exclusion criteria: we excluded younger caregivers below the age of 18, paid caregivers those not involved with day-to-day care provision to older people with serious physical illness.

#### Healthcare professionals

Inclusion criteria: staff working with older people or providing care to older people in primary care which included nurses, medical doctors, clinical officers, health assistants, community health workers and at least 6 months working experience in the setting.

Exclusion criteria: healthcare professionals not working in primary care or not providing care to older people with serious illness and those with less than 6 months working in a primary care setting.

### Recruitment

Clinical staff at study sites reviewed patients’ clinical records to identify potential patient participants. They verbally introduced the study to older people and referred them to the study researchers if they expressed interest in participation.

Patient participants willing to take part in the study were then requested to nominate a caregiver in line with the inclusion criteria. Researchers provided information sheets to patients and caregivers in local languages, and they were allowed to discuss with other family members before they decided whether to take part.

Patient and caregiver information sheets and consent forms were translated by experienced professional translators who are fluent in the local language in each country. Forward and back translation was performed by two translators, and differences were resolved through discussion.

Clinical staff were recruited through facility managers who identified eligible potential participants. They also introduced the study to staff and referred them to the study researchers if they expressed interest in participation. Researchers then provided information sheets to staff, and they were allowed to discuss with their colleagues before they decided whether to take part.

For all participants who expressed willingness to take part, informed consent was administered by study research assistants.

### Sample size

A purposive sampling frame was used to identify and select all participants who were knowledgeable and experienced with primary palliative care services as recipient or service providers,^35^ including their availability, willingness and ability to share experiences and opinions.^36 37^ The anticipated sample size to achieve our study aim was n=45 older patients (n=15 per country) and n=45 (n=15 per country) caregivers. All participants were sampled by age, gender, tribe, diagnosis and country of origin (an additional criterion of relationship to patient was applied to caregivers) to achieve maximum variation in our sample. We estimated that a minimum of 15 interviews for patient and caregiver categories in each country would provide sufficient information power regarding the experience of living with multimorbid illness or caring for an older person with multimorbid illness in relation to study aims.

For staff, we anticipated recruitment of at least 6–12 participants in a focus group discussion, that is, at least 12–24 clinical staff at two primary care sites in each country. Staff were purposively sampled by professional cadre and clinical setting.

### Data collection

For patients and caregivers, a demographic questionnaire was administered to participants, capturing age, gender, education level, employment status, religion, diagnosis and date of diagnosis and relationship with the patient.

The staff demographic questionnaire captured: gender, professional cadre, years of experience working in primary palliative care and years of experience working at the facility.

Some patient and caregiver interviews were conducted at the health facility, while some were conducted at the participants’ homes. Some patients were interviewed in the presence of a family member. All focus groups were conducted at the health facility.

In-depth semistructured qualitative interviews were conducted with patients and caregivers using an iteratively refined topic guide. We explored barriers and facilitators to healthcare, expectations of care and the health system, patient/family/staff interaction, communication and information sharing, decision-making, care engagement, views with clinical consultations, preferred views of person-centred care, current direct medical and non-medical treatment, and care costs plus productivity losses. Data collection continued until we reached data saturation, that is, the point at which we could not identify new themes related to the study aim during the iterative analysis.^38 39^

Staff topic guide explored: patient and family needs (physical, psychological, social and spiritual care) in line with WHO definition of palliative care;^40^ current standard primary care practice for multimorbid older people; potential barriers and facilitators; system changes and training needs to achieve delivery of primary palliative care.

All topic guides (online supplemental file 1) were developed based on our review of reviews appraising models of palliative care to improve quality of life for older people building on key themes and address LMIC gaps^41^ and our review of the evidence for primary palliative care in LMICs.^42^

We audio recorded interviews and focus group discussions using encrypted devices. Interviews took 30–60 min, while focus groups lasted for 90–110 min. We held weekly debrief meetings to discuss interviews and focus group discussions using field notes and reflective forms which were compiled by researchers.

All researchers who collected data have over 5 years’ experience of conducting qualitative research. DK is a male PhD candidate and Lecturer in Palliative Care at the Kamuzu University of Health Sciences and collected data in Malawi. In Zimbabwe, data collection was led by DC, a male palliative care physician with over 10 years’ experience in research and studying for MSc in palliative care at the University of Cape Town. In Ghana, John Amissah, a male PhD candidate at Kwame Nkrumah University of Science and Technology, collected data.

### Data analysis

Audio recordings were transcribed verbatim and translated into English where necessary, pseudonymised and imported to NVIVO 12 pro for framework analysis,^43^ with all participant samples analysed jointly to a single coding frame.

The analysis commenced with familiarisation with the data whereby the researchers read and re-read transcripts. The second phase involved annotating the transcripts using line-by-line coding and identifying initial themes. The researchers then applied these themes to a sample of patient transcripts (n= 2) and caregiver transcripts (n= 2) for each country through a process of indexing the data. The analysis then moved to the charting phase and the creation of a coding framework. This coding frame was shared with the research team from each country (DK, MM and DVB in Malawi; AB in Ghana; and DC and LP in Zimbabwe). Researchers from each country then applied the framework to a further sample of n= 2 transcripts for both patient and caregivers. Researchers added new codes where necessary as coding progressed. All researchers then met to discuss the coding frame, and all differences were resolved through discussion. The team then agreed on the overall coding frame which was used to code all the remaining transcripts. Themes and subthemes were developed with a definition to ensure established internal consistency during coding, and quotes selected for reporting of each code to exemplify meaning with an anonymised ID. All data were analysed jointly to a single coding frame.

This approach enabled us to chart individual responses into a common coding frame, providing easy comparison between sets (ie, patients, caregivers or staff) and identify common themes and divergent cases. The frame also enabled comparison on potential variability between countries and diagnostic groups. ^43^

###  Ethical approval

Ethical committee approval was granted by the Principal Investigator’s Institutional Review Boards of King’s College London (Ref: HR-19/20–18524; online supplemental file 2). Ethics approval was obtained for all local ethics committees such as Ghana Health Service Ethics Review Committee (Ref: GHS-ERC 012/03/21; online supplemental file 3), College of Medicine Research Ethics Committee (in Malawi) (Ref: P.08/20/3108; online supplemental file 4) and Medical Research Council of Zimbabwe (Ref: MRCZ/A/2759; online supplemental file 5). All study participants provided written informed consent for study participation, data analysis and publication.

### Patient and public involvement (PPI)

Prior to this work, there were no formal patient and public involvement (PPI) structures for older people across the three countries. We therefore established an African PPI palliative care network which informed research procedures and dissemination throughout the programme. The project formed community representative groups in each country, and research study sites now have members from community groups.

We held stakeholders’ dissemination conferences in each country at the end of the study. Patients and caregivers were involved in organising and disseminating study findings to policy makers, for example, the Malawian Stroke Association is now co-designing, disseminating and working to implement a pathway for better care as a result of the patient engagement activities.^44^

## Results

### Participant characteristics

A total of 135 individuals participated: Ghana (n=12 patients; n=10 caregivers; n=12 healthcare professionals with n=6 at each of two sites); Malawi (n=15 patient/caregiver dyads; n=22 healthcare professionals with n=11 from each of two sites); and Zimbabwe (n=14 patients, n=12 caregivers and n=23 healthcare professionals with 12 and 11 participants at two sites).

The majority of the patients were female and Christians with secondary school education, not employed and living with hypertension and diabetes. Caregivers were mostly females with secondary education, unemployed and Christian, and were siblings of the patient participant.

For staff, the majority were female nurses, with over 7 years’ working at the facility and 10 years’ work experience (see online supplemental table 1).

### Main findings

**Figure 1:**
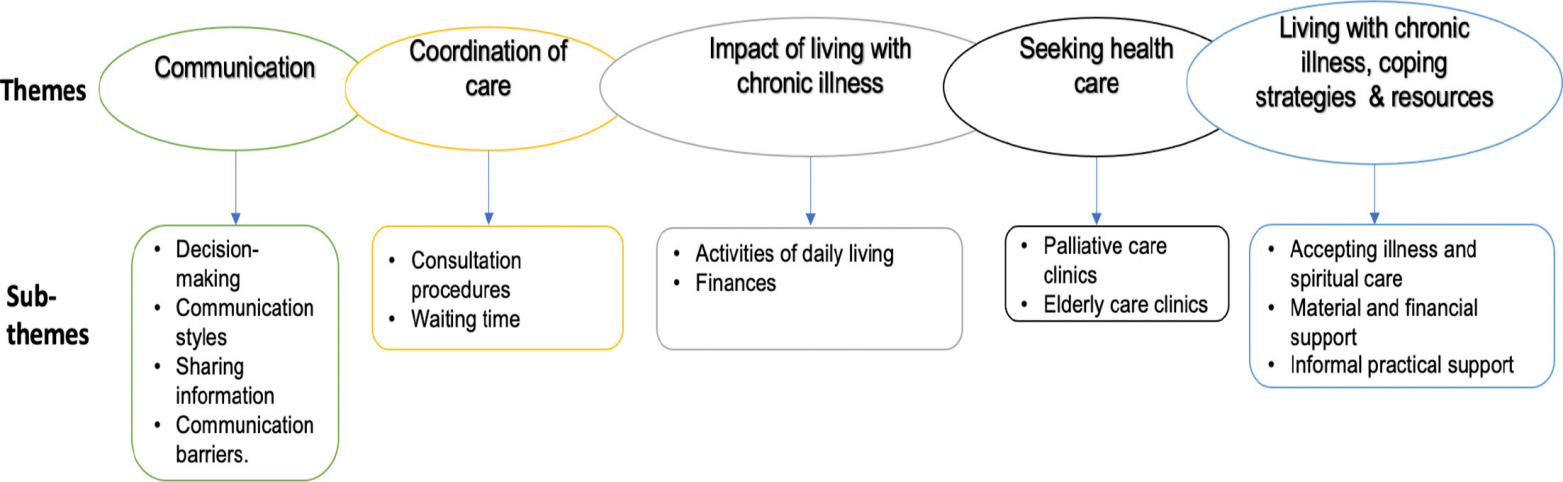
Five main themes identified in the study: (1) communication, (2) coordination of care, (3) impact of living with chronic illness, (4) seeking healthcare and (5) coping strategies. Each of the five themes has subthemes underneath it.

The analysis revealed five main themes: (1) communication, (2) coordination of care, (3) impact of living with chronic illness, (4) seeking healthcare and (5) living with chronic illness: coping strategies and resources. Each theme is explained below, and supporting quotes are presented in online supplemental table 2. Figure 1 illustrates the themes and subthemes and how they interlink.

### Theme 1: communication

The four subthemes within communication were: (a) decision-making, (b) communication styles, (c) sharing information and (d) communication barriers. Each is described below.

#### 1a) Decision-making

Across all the three countries, family members were crucial in terms of decision-making for older relatives. They made decisions about when and where to receive treatment. Sometimes there were disagreements about decisions made by older people, especially if they felt that the patient lacked capacity to make informed decisions or proper decisions for themselves due to illness impact, for example, cognitive function (quote 1). On some occasions, disagreements occurred between caregivers and patients, for example, patients declining hospital attendance because of ‘not wanting to bother’ health professionals. Patients expressed that frequent hospital visits may make some clinicians ‘unhappy’ by seemingly poorly managing the person’s care (quote 2). Family caregivers across all settings appreciated when healthcare professionals involved them in decision-making, creating a sense of feeling valued (quote 3).

#### 1b) Communication styles

Patients and caregivers valued being treated by health professionals with dignity and respect, for example, being greeted and addressed by their name and recognising their presence. Patients and caregivers valued those health professionals that gave them ‘hope’ and belief that they would survive (quotes 4–5). Conversely, they also reported some healthcare professionals not bothering to greet them and allow them to voice concerns (quote 6). Concomitantly, healthcare professionals recognised how their attitude and behaviour towards patients and their families/caregivers affected the way patients engaged with them.

#### 1c) Information provision

Participants reported that some healthcare professionals did not disclose the diagnosis to the patient and/or the family; they wondered why this was the case after several clinical contacts with the same healthcare providers (quote 7). Interestingly, across all the settings, others felt well-informed about their diagnosis, causes and common symptoms (quote 8).

Across all three countries, patients and caregivers reported that they rarely or never received printed resources such as leaflets. Although healthcare professionals provided patients with verbal information during clinical appointments, participants felt that printed resources are important to reiterate verbal information.

Healthcare professionals observed that despite information provision, patients often seemed little to understand their diagnosis, even after living with a chronic illness for several months. They acknowledged that detail could be insufficient or poorly understood, and the importance of repeating information at each visit, treating patients as if they had not been informed fully about their illness before (quote 9). In some cases, patients and/or caregiver participants reported that they were seemingly ill-prepared or unwilling to receive ‘bad news’.

#### 1d) Communication barriers

Healthcare professionals identified that patients were more able to communicate their problems if they were given space and a conducive environment. However, there were multiple communication barriers to achieving this. In most settings across the countries, there was often inadequate space to discuss sensitive issues with the patient. Patients and caregivers typically waited many hours to be seen in the clinic due to long queues. Moreover, the consultation was often rushed with the patient and/or family having insufficient time to receive and discuss detailed information with the healthcare professionals. Health professionals felt that patients were often unable to provide full information about their illness, for example, history and presentation of their symptoms, medication history and side effects, for them to complete a clinical assessment and understand the problems and concerns and management. This was both due to illness and fatigue after waiting many hours to be seen. Patients were not pro-active in raising their concerns and problems, rather preferring to wait for the health professional to ask (quote 10). The use of technical language by health professionals further impeded the understanding for patients and families about the information being asked for and given. Language barriers were reported (especially in Ghana and Malawi where health professionals could not speak the local language). In such situations, healthcare professionals relied on family members to translate or other members of staff who were familiar with the patient’s language (quote 11).

### Theme 2: care coordination

Patient and family caregiver participants reported that in general, there was good coordination of care and consultation between healthcare staff within the clinical team and across services (quotes 12 and 13). In Malawi, participants identified problems in coordination between investigation, prescribing and dispensing with limited availability of many prescribed medicines (quote 14). Health professionals also observed how the behaviour of colleagues affected the delivery and coordination of care, with, for example, staff absenteeism increasing workloads for colleagues. Moreover, patients reported how the rationale for consultation was not always clear and they were often not invited to participate in discussion, with healthcare professionals—speaking mainly to the family, giving information but little consulting with the patient. This impeded sharing their concerns and receipt of care and treatment aligned with their priorities.

Patients and family caregiver participants expressed the need for healthcare professionals to attend to them on time and treat them with urgency. They stated that they used to come to the hospital very early, but it took time for them to be attended to. This was not only due to long queues, but because some of the health professionals reported late for work; hence, some patients were waiting to be attended to while struggling with pain (quotes 15–16).

### Theme 3: impact of illness

Two main subthemes were reported under the impact of illness: (a) activities of daily living and (b) finances.

#### 3a) Activities of daily living

Patients reported that multimorbid serious illness had devastating effects on their daily lives, including restricted diet, participation in religious activities, and family or community events (eg, because of urinary incontinence, use of a stoma bag, reduced mobility, chemotherapy causing fatigue) (quote 17). This was very frustrating to many older people who lost opportunities to engage with other community members. Illness such as stroke affected day-to-day home activities, for example, farming for income and food source.

The majority of participants reported having inadequate food to meet their needs, compounded by not being actively involved in income-generating activities (quote 18).

#### 3b) Finances

Older people experienced catastrophic costs due to illness. Having two or more chronic conditions required regular monthly or 3 monthly hospital visits for follow-up care or to collect medication. Even though these were public hospitals, medication and supplies were often limited. This required patients to buy medicines privately and pay out-of-pocket. Most did not have health insurance or had insufficient insurance cover for the required laboratory investigations, medications and supplies. Most of the patients and caregivers lived in remote rural communities, far away from the healthcare facilities requiring a taxi or other means of transportation to reach the hospital in the main town, often necessitating many hours of travel and cost (quote 19).

Older people found it difficult to balance costs of medication and daily living costs such as food and school fees. They valued the education of their children, and some patients chose to pay for their children’s school fees at the expense of paying for health services. Some participants borrowed money from friends, relatives and workmates to meet the costs of treatment (quote 20). Finance was a challenge for older people because they could not generate enough income to pay for their medical costs with limited/no opportunities for paid employment/farming due to illness or following retirement with no/little state pension provision. There were a few older people who generated income by moving back to their village to stay in a second home and renting their home in the city to raise income for their care and treatment (Quote 21).

Patients needed medication, medical supplies (eg, stoma bags) and mobility aids (wheelchairs). Mobility challenges often meant they were unable to visit friends or attend community social activities (quotes 22).

### Theme 4: seeking healthcare

There were no dedicated clinics for elderly people in Malawi or Zimbabwe. Ghana had a geriatric clinic for people aged ≥65 years held on a specific day (quote 23). In Malawi and Zimbabwe, there were specific days/clinics for chronic illnesses (eg, hypertension, diabetes), and patients were booked according to their diagnosis (quote 24). All the settings in each country held a specific palliative care clinic.

The lack of clinics for older people in Malawi and Zimbabwe meant waiting in long queues, posing specific challenges, for example, diabetic patients waiting for fasting blood tests. Participants were required to wait in up to four different queues: (1) to have vital signs checked, (2) to have blood glucose checked, (3) consultation with the medical doctor and (4) to collect medication. Older people felt that the system needed to have a queue for ‘elders’ to minimise the wait time. In some cases, across the countries, older people are prioritised, but this is not institutional policy. Healthcare professionals would sometimes collect medication for the patient or speak with the others in the queue to allow an older person to be assisted first (Quote 25).

### Theme 5: living with chronic illness, coping strategies and resources

We identified three subthemes underneath this theme: (a) accepting illness and spiritual care, (b) material and financial support and (c) informal practical support and care.

#### 5a) Accepting the illness and spiritual care

Participants typically reported accepting their diagnoses as ‘normal ageing’ and preferred this approach to disclosing that they were living with chronic illness to, for example, members of their village. Participants reported feeling better about their health by comparing themselves favourably to patients with a seemingly poorer health status (Quotes 26–27).

Spiritual strategies to cope with illness were commonly reported across the countries. Spiritual support was accessed at home from religious and spiritual leaders whose guidance helped reduce worry through prayer, holy communion, singing songs of hope and receiving words of encouragement that uplifted their spirits (quote 28).

#### 5b) Material and financial support

Participants received a great deal of support from their families and friends, including money to buy food, medications and medical supplies. Food was very important, especially among patients who were on specific restricted diets which they could not afford (quote 29).

Family and friends also paid hospital bills for older people or paid for their health insurance.

They also received food items from the church, community members and neighbours. Some patients were able to continue to grow crops and rear animals to sell to fund living expenses and/or medical costs (quote 30).

#### 5c) Informal practical support and care

Older people were heavily reliant on, and greatly valued, the informal care provided by family, friends and community members. This encompassed a breadth of practical support, such as cooking, fetching water, cleaning the house, gardening, farming assistance (cultivating land, planting maize, harvesting) and financing the hire of manual farming labourers, for example, for fertiliser application and harvesting (quote 31). The patients often relied on family and friends to escort them for hospital appointments and collect medication from the hospital on their behalf (this reduced patient costs for transport and conserved energy). In some cases, community members paid for transport/hired a vehicle or used their own vehicles to provide free transport (quote 32). Caregivers also provided nursing care such as wound dressing, changing stoma/ostomy bag, assisting with eating and drinking, medication management and personal hygiene.

## Discussion

The WHO guidance on integration of palliative care into primary healthcare states that services must be developed that are responsive to local needs and contexts.^21^ The significance of this data is that it offers rigorous and novel evidence to fulfil the goals of the WHO primary palliative care vision.

The guidance focuses on enabling policy, drug availability and training. Our data demonstrate that drug availability is a challenge for older multimorbid people in the community. The data also specify the type of training to be delivered, particularly on involving and supporting patients and families in decision-making. This is consistent with findings that when patients feel comfortable communicating their problems and concerns and seeking appropriate support; they report lower levels of anxiety and stress.^45^ Patients who are involved in their own care also report a safer care experience.^46^

Promoting engagement of older multimorbid people in their social and community networks (eg, religious activities) should also be encouraged. Our data found community members and neighbours to be instrumental in providing practical support such as providing transport, assisting with farming and gardening, which in turn improved access to food. Although these ‘public health’ approaches to supporting people to die well in the community offer improved outcomes, currently little evidence exists from LMICs.^47^ Such approaches can also support family caregivers. Our data reveal the crucial roles they perform for older multimorbid people, including personal care, practical and financial support (eg, giving money for transport, medication and supplies including insurance and farming supplies).

The need to invest in health and social support for older people in particular those living in LMICs is a priority.^48^ The original outputs of this study also support the WHO Healthy Ageing 2021–2030 policy, which calls for collaborative action to improve the lives of older people, families and communities through delivery of person-centred integrated care and primary health services.^49^

The ‘2020 vision’ for American healthcare^50^ advocates for automatic and affordable health insurance for all, access to care, patient-centred care, information-driven care that is based on scientific evidence and supported by clinical information systems, and commitment to quality improvement and betterment of health outcomes by everyone in the healthcare sector. Our findings show that patients and families faced financial challenges to pay for their health services. Even though public services are free, in most cases, medication and supplies were out of stock which meant patients and families had to pay for these services. Some patients had medical insurance, but this was not enough to sustain the costs of care, and most of the patients had already used their insurance cover.

A systematic review of the evidence underpinning the concept of person-centred care for adults with serious physical illness identified being treated with dignity, being respected and valued as core values and practices.^51^ However, the data were exclusively from high-income countries. Therefore, this study provides originality in generating primary data in LMICs health systems and populations to inform person-centred health services. It is notable that our data identified similar themes to the review, suggesting the presence of some universal activities and values in person-centred healthcare.

Our previous qualitative study on developing a global practice-based framework of person-centred care was conducted in middle-income countries.^52^ It concluded that person-centred care requires particular structural features of the healthcare system to be in place, such as professional education in value-based care and collaborative partnerships with community health workers. The data presented in this study suggest that aspects of person-centred care can be provided within primary care using available resources. For example, older people reported that they need to be prioritised when accessing care or need to have dedicated clinics that do not require repeated long queues. These needs do not necessarily require infrastructure adjustments but rather policy change and adjustments within the existing facility and its pathways.

A future challenge for this work will be to measure person-centredness and outcomes from new models of person-centred care for multimorbid older people. The views of patients and caregivers in this study are in line with aspects of the eight dimensions of *patient-centred care* as defined by the Picker Institute which include (1) respect for the patient’s values, preferences and expressed needs; (2) information and education; (3) access to care; (4) emotional support to relieve fear and anxiety; (5) involvement of family and friends; (6) continuity and secure transition between healthcare settings; (7) physical comfort; and (8) coordination of care.^53^ Appraisal of face and content validity to ensure any tool captures the specific needs of this population and the goals of palliative care will be an important initial step towards valid, reliable and responsive measures.

This study demonstrates the need for better integration of the assessment and delivery of medical, nursing, allied health professional and social services for older people to achieve person-centred care. This has great potential benefit as integrated services can minimise costs.^54^ For example, scheduling an appointment that enables a holistic person-centred assessment (of physical, social, psychological and spiritual needs) during a single visit offers benefits to health systems as well as patients and families. Research is needed on what adaptations of the home environment can be made to enable older people to remain socially connected and an integral part of their communities. These findings also call for researchers to design context-appropriate needs-driven interventions to improve the well-being of older people.

Research is also needed to inform the development of policy and for prioritisation of health systems and primary care services to address the medical and psychosocial needs of older people in LMICs. Feasibility studies and full trial to evaluate the effectiveness of integrated palliative care interventions for older people are needed, including implementation strategies and sustainability of such interventions in these settings. This work also informs the need to develop methodological guidance for primary palliative care trials in LMIC and intervention manual adaptation to partner countries.

Data collection was conducted at district hospitals which are the largest providers of primary healthcare services, and they also serve as referral facilities. In our recent review, we identified five models of primary palliative care in LMICs: (1) palliative care delivered by multidisciplinary teams of generalists (nurses, physicians, community health workers, social workers, physiotherapists and pharmacists) in primary care clinic;, (2) multidisciplinary teams of generalists deliver palliative care services in people’s homes; (3) community volunteers provide home-based care including referral of complex cases to healthcare facilities; (4) generalists, including family physicians, general practitioners and hospitals, deliver palliative care services based in hospitals and other secondary or tertiary healthcare settings; and 5) palliative care specialists visit hospitals to provide palliative care on specific days.^13^ All these models are available in all the three countries; however, the volunteer model is only available in Malawi and Zimbabwe.

We have maximised the rigour of our study by sampling across three stakeholder populations, with cross-national data collection and analysis. This is highly original, in that work to date, has not identified cross-national feasible and acceptable ways to proceed while recognising national differences. This is highly significant as we seek ways to realise UHC palliative care goals for ageing populations.

### Strengths and limitations

A major strength of our study is that we have moved the evidence base for primary palliative care in LMICs forward, with data from healthcare professionals, patients and families from three low-income countries. Data were analysed by the research team from all the three countries which enhanced the rigour of the findings. We used purposive sampling to recruit study participants across all settings, and we achieved a diverse sample. Local researchers led data collection and were fully engaged in the development of the coding frame. Although differences between countries were not evident in the analysis, we would anticipate that context will have an important influence in the subsequent development and implementation of models of primary palliative care for older people. This has enabled us to advance research in palliative care for older people (previous palliative care research in Africa had been almost exclusively cancer and/or HIV^11^) and in primary palliative care in LMICs, which had not previously addressed the needs of older people.^42^ This is the first study to explore multiple stakeholders’ perspectives on a model of care suitable for older people in primary palliative care settings from three different LMICs.

This study had several limitations. Senior local researchers in Ghana and Malawi did not speak the local languages in which researchers had conducted the interviews. Therefore, an independent language expert translated the topic guide and data. Some subtleties in meaning could potentially have been lost during this process. However, to mitigate this, the research assistants who conducted the interviews were consulted in the analysis process. Some patient interviews were conducted in the presence of the caregiver; this might have limited the patient’s ability to verbalise all the problems and concerns. However, we did not identify any inconsistencies between caregiver and patient accounts when the dyad was interviewed separately.

## Conclusions

Older people frequently use primary palliative care settings for treatment and care due to problems and concerns which they experience. These require a person-centred palliative care approach, involving both patient, families and community members. Staff working in primary care should value the social network of patients and families to promote quality of life and make structural adjustments within their settings to improve patients’ and caregivers’ experience interacting with the healthcare system.

This study has identified the needs of a population that has been overlooked in previous palliative care research, in a setting (ie, primary care) that offers great potential to improve access to timely care. The literature review and this primary data will now be used to develop and evaluate novel context-specific models of care. The significance of this work will further support implementation of the WHO guidance on primary palliative care.

## Supplementary material

10.1136/bmjph-2024-001355 online supplemental file 1

10.1136/bmjph-2024-001355 online supplemental file 2

10.1136/bmjph-2024-001355 online supplemental file 3

10.1136/bmjph-2024-001355 online supplemental file 4

10.1136/bmjph-2024-001355 online supplemental file 5

10.1136/bmjph-2024-001355 online supplemental file 6

10.1136/bmjph-2024-001355 online supplemental file 7

## Data Availability

Data are available upon reasonable request.
