# Supplementary material for: Integrating palliative care into primary care for older people with multimorbid serious illness: a multinational qualitative cross-sectional study in Sub-Saharan Africa
Source: BMJ Public Health. 2025 Mar 23;3(1):e001355. doi: 10.1136/bmjph-2024-001355 (PMC11934398; doi:10.1136/bmjph-2024-001355)
Supplement: online supplemental file 1 [file bmjph-3-1-s001.docx]

**TOPIC GUIDE FOR IN-DEPTH INTERVIEWS WITH PATIENT**

| **SECTION 1: About the patient and Illness**  Could you tell me a bit about yourself?  Would you mind telling me a little bit about when you first became unwell? |
| --- |

| **SECTION 2:** Please could you tell me about **living with your disease** – On a day-to-day basis, what are your main needs, priorities and concerns? |
| --- |
| 1. How does your illness affect you on a day to day basis?    1. **your body:** pain, breathlessness, fatigue and other physical symptoms)    2. **psychologically?** How you feel in yourself, How able are you to cope with your illness?    3. **socially** and what you can do day-to-day?    4. How does your illness affect your religion/spirituality? What are your **religious or spiritual** needs, if any? 2. What would you say are your **main priorities** at the moment? Are there certain aspects of your life or your health that are most important to you at the moment? 3. How do you think your illness will affect you in the future? Do you have any particular concerns or worries about the future?   **SECTION 4:** Your feelings about the care you receive |

| **SECTION 3:** Now thinking about the problems you have described, I’d like us to talk about what happens when you see someone at a [health centre]? |
| --- |
| 1. Do the health care providers ask about your concerns? Why?    1. Do you discuss your concerns? Why? 2. Do you feel health professionals know what matters to you and what is important to you? Do you feel the care you are given focuses on what is important to you? Why? 3. Could you tell me about whether the **people managing your care listen to you and understand your needs and concerns** and those of your **family/friends?**    1. Explain why yes or no |

1. What do you think about the place where you receive care- is it a nice place? What do you like or not like?
2. What kind of **support** did you receive there?
3. When you go to the health facility **how are you treated as an individual**?
4. How do care staff behave towards you?
5. How would you like them to behave towards you?
6. What things should doctors and nurses do to better understand you as a person?
7. What would you like them to ask you about and know about you?
8. What could be done to make sure that the people that deliver your care interact with you [in the ways that you want]?
9. How have you found the **communication between the team** of people managing your care? How have they worked together as a team?
10. What do you think about the way in which any **information has been given to you** **about your disease and care**?
    1. Have you had the opportunity to ask questions?
    2. Have you felt able to ask the questions that are most important to you?
    3. If not, why?
    4. Do you get the answers and information that you want?
    5. What would you like to know about your disease and care?
    6. How would you like that information to be shared with you?
    7. Is there anything you would **not** want to know about your disease and care?
    8. Is information given to you in a way that you could understand?

**SECTION 5**: Your family and making decisions

1. Have your **family/friends** been given **information – have they** been involved in your appointments?
2. How involved would you like them to be?
3. When there are any decisions or choices to be made about your care, who **is involved in that decision making**?
   1. E.g. you the patient, family

d) What sort of things do you do at home to try and **manage your own health** e.g. if you are in pain, or breathing problems- what do you do to make yourself feel better?

- 1. What might help you to take care of yourself/self-manage your illness at home?
  2. Information, phoneline, written exercises etc
  3. How could things be made easier for you to be cared for at home?

e) Do you ever go to the **emergency room** at the hospital?

- 1. How do you decide to go there?
  2. Who makes the decision?
  3. Who would you like to be involved in that decision making?

f) Could you tell me about how your **different health appointments** are scheduled?

- 1. Where has your care been provided – where have you had to go?
  2. How often do you go to the community health clinic/district hospital?
  3. How do you decide when to go?
  4. How would you like your appointments to be arranged- how could it be better for you?

g) Has anyone ever talked to you about how your care needs might change over time, **and about your future wishes and priorities for care**?

- 1. Would you want to have that discussion about your care in the future?
     - why?

**SECTION 6: Services and costs**

| 1. What services are provided to you at this facility when you come to seek help? (probe: laboratory tests, radiology etc). How much do you pay for these services? (probe for each of the services mentioned) 2. Are there specific days or special clinic days for the elderly with multimorbid serious illness? Which are these days? How often do you visit the clinic? monthly, every three months? every six months? Are these on appointment basis or visits you make when you are feeling unwell? What are the main issues/reasons that make you visit the facility? 3. What type of drugs are you taking? How often do you take these drugs? What are the costs of the drugs, what are the costs of diagnostic tests? How often are these diagnostic tests conducted? Who pays for these costs? How do you travel to access the services or to visit facility? (probe Ambulance, taxi, own transport? who pays for transport services) 4. Are there any other services you seek for your illness elsewhere other than at this facility (probe: traditional care, counselling, complementary services), How much do these services cost? 5. What do you think might be the best possible way for health care professionals to give care to elderly patients with multimorbid serious illness when they visit the health facility? 6. What would you change or do differently? |
| --- |

**TOPIC GUIDE FOR IN-DEPTH INTERVIEWS WITH CAREGIVERS**

| **SECTION 1: About you and your patient**  Could you tell me a bit about yourself?  Please tell me a little bit about when your relative first became unwell? |
| --- |

| **Section 2:** Please could you tell me about your current experience of having an elderly relative with multimorbid serious illness? On a day-to-day basis, what are your relatives main needs, priorities and concerns? |
| --- |
| 1. How does serious illness affect you on a day to day basis?    1. **your body:** pain, breathlessness, fatigue and other physical symptoms)    2. **psychologically?** How you feel in yourself, How able are you to cope with your illness?    3. **socially** and what you can do day-to-day?    4. How does their illness affect your religion/spirituality? What are your **religious or spiritual** needs, if any? 2. What would you say are your **main priorities** at the moment? Are there certain aspects of your life or your health that are most important to you at the moment? 3. How do you think the illness will affect you in the future? Do you have any particular concerns or worries about the future? |

| **Section 3:** Now thinking about the problems you have described - I’d like us to talk about what happens when you see someone at a [health centre]? |
| --- |
| 1. Do the health care providers ask about your concerns? Why?    1. Do you discuss your concerns? Why? 2. Do you feel health professionals know what matters to you and what is important to you? Do you feel the care you are given focuses on what is important to you and/or the patient? Why? 3. Could you tell me about whether the **people managing your patient listen to you and understand your needs and concerns** and those of your **patient?**    1. Explain why yes or no |

**Section 4:** Your feelings about the care you and/or your family receive

1. What do you think about the place where you receive care- is it a nice place? What do you like or not like?
2. What kind of **support** did you receive here/there?
3. When you are/go to the health facility **how are you treated as an individual**?
4. How do care staff behave towards you?
5. How would you like them to behave towards you?
6. What things should doctors and nurses do to better understand you/your patient as a person?
7. What would you like them to ask you about and know about you?
8. What could be done to make sure that the people that deliver care interact with you [in the ways that you want]?
9. How have you found the **communication between the team** of people managing your care? How have they worked together as a team?
10. What do you think about the way in which any **information has been given to you** **about your disease and care**?
    1. Have you had the opportunity to ask questions?
    2. Have you felt able to ask the questions that are most important to you?
    3. If not, why?
    4. Do you get the answers and information that you want?
    5. What would you like to know about your disease and care?
    6. How would you like that information to be shared with you?
    7. Is there anything you would **not** want to know about your disease and care?

**Section 5:** Family and decision making

1. Have you or your **patient** been given **information – have they**  involved you in your patient's appointments?
2. How involved would you like them to be?
3. When there are any decisions or choices to be made about your patient's care, who **is involved in that decision making**?
   1. E.g. the patient, family, friends
4. What sort of things do you do at home to try and **manage your patient's health** e.g. if they are in pain, or breathing problems- what do you do to make the patient feel better?
   1. What might help you to take care of your patient/self-manage your patient's illness at home?
   2. Information, phoneline, written exercises etc
   3. How could things be made easier for you to care for your patient at home?
5. Do you ever go to the **emergency room** at the hospital?
   1. How do you decide to go there?
   2. Who makes the decision?
   3. Who would you like to be involved in that decision making?
6. Could you tell me about how your patient's **different health appointments** are scheduled?
   1. Where has your patient's care been provided – where have you had to go?
   2. How often do you go to the community health clinic/district hospital with the patient?
   3. How do you decide when to go?
   4. How would you like your patient's appointments to be arranged- how could it be better for you?
7. Has anyone ever talked to you about how your patient's care needs might change over time, **and about your patient's future wishes and priorities for care**?
   1. Would you want to have that discussion about your care in the future?

why?

**Section 6:** Services and costs

| 1. What services are provided to your patient at this facility when you come to seek help with your patient? (probe: laboratory tests, radiology etc). How much do you pay for these services? (probe for each of the services mentioned) 2. Are there specific days or special clinic days for the elderly with multimorbid serious illness?    1. Which are these days?    2. How often do you visit the clinic? (probe: monthly, every three months? every six months)?    3. Are these on appointment basis or visits you make when your patient is feeling unwell?    4. What are the main issues/reasons that make you visit the facility? 3. What type of drugs does your patient take?    1. How often do you take these drugs?    2. What are the costs of the drugs?    3. What are the costs of diagnostic tests?    4. How often are these diagnostic tests conducted?    5. Who pays for these costs (probe drug costs and diagnostic costs)?    6. How do you travel to access the services or to visit facility? (probe Ambulance, taxi, own transport? Who pays for transport services)? 4. Are there any other services you seek for your patient's illness elsewhere other than at this facility (probe: traditional care, counselling, complementary services), How much do these services cost? 5. What do you think might be the best possible way for health care professionals to give care to elderly patients with multimorbid serious illness when they visit the health facility? 6. What would you change or do differently? |
| --- |

**TOPIC GUIDE FOR FOCUS GROUP DISCUSSION**

| **Section 1: About yourself**  Could you tell me a bit about yourself?  Would you mind telling me a little bit about your role in caring for elderly people with multimorbid serious illness such as heart failure, COPD, Cancer, HIV/AIDS? |
| --- |

| **Section 2:** Please could you tell me about your experiences of caring for elderly patients with multimorbid serious illness. On a day-to-day basis, what are patient’s and families’ main needs, priorities and concerns? |
| --- |
| 1. How does the illness affect them on a day to day basis? How does it affect them **physically?**  How does it affect them **psychologically?** 2. What are the effects on their life **socially?** 3. How about their **religious or spiritual** wellbeing – is this affected by their illness? 4. d) How do patients/families talk about their illness affecting them in the future? Do they have any particular concerns or worries about the future? |

| **Section 3:** [Referring back to any specific points raised above] - In what ways are [these needs] being met by the health services? In what ways are they not being met? |
| --- |
| 1. In what ways do you think patient’s **needs** (physical/psychological/social/religious/ spiritual) are **assessed**? In what way are these needs **being met**? if not why not, if yes then how? 2. What are things the older people with serious illness **value in life (personal, health, moral values)** Are these things being assessed and met? In what ways are they not? Are they well managed?   Do you tend to know about your patients in terms of their personal values and what matter to them? Do your patients share information about their relationships and who matters to them? How about for people in same sex relationships – would this be information that you would know? Do you ask people about their sexual orientation? How are people who are LGBT supported in your services? Are there particular challenges for these individuals? If so, what?   1. What could help you to manage these needs better? How about training and support, and mentorship is there anything that would be useful? |

1. What is the current **physical caring environment** like at the district hospital (health facility)?
2. Do you think the caring environment could be improved? How? How could these changes be made?
3. What are the practical challenges you experience that affect the provision or delivery of care to the elderly with multimorbid serious illness?
4. To what extent is that care received by the elderly with multimorbid serious illness **individualised**?
5. What things should clinicians ask to better understand older patients with multimorbid serious illness as an individual?
6. What could we do to make sure that patients feel respected by the clinicians? What would make these patients feel like they are treated with dignity?
7. How do you currently **communicate and interact with older patients with serious illness?** What about their **family/friends**?
8. What do you ask them and their family/friends about?
9. What problems do you expect them to talk about at appointments?
10. Are there any problems that you don’t expect them to talk about?
11. What are the most important things that doctors and nurses should know about elderly patients with serious illness to make care as good as can be?
12. What do you think would be most helpful for clinicians to ask patients to help you really understand what matters to them?
13. Do you think you know that already about your patients? Why?
14. What don’t we ask at the moment?
15. Why do we think we don’t ask those questions?
16. How easily do you feel able to coordinate the care that you provide by working and communicating with your colleagues?
17. How easy is it to keep everybody informed? In what ways? Why?
18. Are there any ways in which you think you could be better coordinated as a team?
19. How could you be supported to make that happen?
20. What information do you give to the elderly patients with multimorbid serious illness **about their condition and care**?
    1. How do you provide that information?
    2. What questions do the elderly patients ask?
21. Do you think older patients with multimorbid serious illness and their families get the information that they want?
    1. Is there anything you would **not** want to tell them about their COPD/heart disease/cancer? Why?
    2. Is there any information that it is difficult to provide? How could that be made easier?
22. How much **information do you give to older patients and family/friends** with multimorbid serious illness and to what extent are they involved?
23. How involved do you think they should be?
24. How would it be possible to involve them more?

| **Section 4: Environment, communication, and care coordination** |
| --- |

| **Section 5: Decision making, self-management, services and costs** |
| --- |

1. When there are any decisions or choices to be made about a patient’s care, who **is involved in that decision making**?
   1. To what degree is the patient involved in those decisions? Why?
   2. Why might it be good/bad to further involve them?
   3. How could we help patients to feel part of the decisions that are made about their care?
2. What’s the **standard care and support** delivered for patients (who they see, how often, referrals pathways, communication with emergency/respiratory medicine)?
3. How is this care **planned and coordinated**?
4. What works well/not so well?
5. How do you think this could be made better? What do you think would help you better provide for patient’s needs?
6. Do you/your colleagues talk to patients or family/relatives about prognosis of their illness? Or about the risk of unexpected death during for example stroke attack, heart attack? Or how their care needs might change over time? Why?
   1. What might you need to help you have these conversations?
7. How much are the patients **in control of managing their own health (Self-management)**?
   1. What do some of your patients do themselves to manage any health problems?
   2. What do you think about the idea of finding ways to help patients and families to better manage themselves at home (self-management)?
   3. How could we help them to self-manage their health?
   4. What would be good/bad about that?
   5. How could we do that?
   6. What could we say or do to make patients feel more in control of their own health?
   7. What might facilitate self-management among the older patients at home?
   8. What are the barriers to self-management among the elderly people with serious illness?
8. What services do you provide for the elderly people with serious illness at this facility (laboratory tests, etc).
9. Which days are special clinic days for the elderly with multimorbid serious illness?
10. How often do these patients visit the clinic? monthly, every three months? every six months?
11. What are the costs of drugs, diagnostic tests?
    1. Who pays for these costs?
    2. How do elderly patients travel to access the services or to visit facility? (probe Ambulance, taxi, own transport? who pays for transport services)

| **Section 6: Areas for improvement**   1. What do you think might be the best possible way for health care professionals to give care to elderly patients with multimorbid serious illness when they visit the health facility?   What changes to how the care is delivered would make the biggest differences for patients attending the health facility?  If you could change one thing, what would you change or do differently? |
| --- |
