# Supplementary material for: Integrating palliative care into primary care for older people with multimorbid serious illness: a multinational qualitative cross-sectional study in Sub-Saharan Africa
Source: BMJ Public Health. 2025 Mar 23;3(1):e001355. doi: 10.1136/bmjph-2024-001355 (PMC11934398; doi:10.1136/bmjph-2024-001355)
Supplement: online supplemental file 2 [file bmjph-3-1-s002.pdf]

Research Ethics
Office

Franklin Wilkins Building
5.9 Waterloo Bridge Wing
Waterloo Road
London SE1 9NH
Telephone 020 7848 4020/4070/4077
rec@kcl.ac.uk

KING'S College LONDON

Kennedy Nkhoma

21 July 2020

Dear Kennedy,

Project Title: MAP-care: Multimorbid Ageing Primary Palliative Care in Ghana, Malawi and Zimbabwe.
Project Reference:HR-19/20-18524

I am pleased to inform you that full approval for your project has been granted by the PNM Research Ethics Subcommittee .

Important coronavirus update: In light of the COVID-19 pandemic, the College Research Ethics Committee has temporarily suspended all primary data collection involving face to face participant interactions until further notice. **Ethical clearance for this project is granted. However, the clearance outlined in the attached letter is contingent on your adherence to the latest College measures when conducting your research.** Please do not commence data collection until you have carefully reviewed the update and made any necessary project changes:

https://internal.kcl.ac.uk/innovation/research/ethics/applications/COVID-19-Update-for-Researchers

For your information, ethical approval has been granted for 3 years from 21 July 2020. If you need approval beyond this point, you will need to apply for an extension at least two weeks before this. You will be required to explain the reasons for the extension. However, you will not need to submit a full re-application unless the protocol has changed.

Ethical approval is required to cover the data-collection phase of the study. This will be until the date specified in this letter. However, you do not need ethical approval to cover subsequent data analysis or publication of the results.

Please ensure that you follow the guidelines for good research practice as laid out in UKRIO's Code of Practice for research: http://ukrio.org/publications/code-of-practice-for-research/.

If you do not start the project within three months of this letter, please contact the Research Ethics Office.

Please note that you will be required to obtain approval to modify the study.This also encompasses extensions to periods of approval. Please refer to the URL below for further guidance about the process:

https://internal.kcl.ac.uk/innovation/research/ethics/applications/modifications.aspx

Please would you also note that we may, for the purposes of audit, contact you from time to time to ascertain the status of your research.

If you have any query about any aspect of this ethical approval, please contact the Research Ethics Office:

(https://internal.kcl.ac.uk/innovation/research/ethics/contact.aspx)

We wish you every success with this work.

Yours sincerely,

Mr James Patterson
Senior Research Ethics Officer

For and on behalf of the PNM Research Ethics Subcommittee
