# Supplementary material for: Integrating palliative care into primary care for older people with multimorbid serious illness: a multinational qualitative cross-sectional study in Sub-Saharan Africa
Source: BMJ Public Health. 2025 Mar 23;3(1):e001355. doi: 10.1136/bmjph-2024-001355 (PMC11934398; doi:10.1136/bmjph-2024-001355)
Supplement: online supplemental file 3 [file bmjph-3-1-s003.pdf]

GHANA HEALTH SERVICE ETHICS REVIEW COMMITTEE

In case of reply the number and date of this Letter should be quoted.

My Ref. GHS/RDD/ERC/Admin/App |21|286
Your Ref. No.

Research & Development Division
Ghana Health Service
P. O. Box MB 190
Accra
Digital Address: GA-050-3303
Mob: +233-50-3539896
Tel: +233-302-681109
Fax + 233-302-685424
Email: ethics.research@ghsmail.org
21st July, 2021

Prof Richard Harding
Department of Palliative Care, Policy and Rehabilitation,
Cicely Saunders Institute, King's College London,
Denmark Hill Campus,
SE5 9JP, London

The Ghana Health Service Ethics Review Committee has reviewed and given approval for the implementation of your Study Protocol.

GHS-ERC Number	**GHS-ERC: 012/03/21**
Study Title	Multimorbid Ageing Primary Palliative Care in Ghana, Malawi and Zimbabwe (MAP-Care)
Approval Date	21st July, 2021
Expiry Date	20th July, 2022
GHS-ERC Decision	**Approved**

This approval requires the following from the Principal Investigator

- Submission of a yearly progress report of the study to the Ethics Review Committee (ERC)
- Renewal of ethical approval if the study lasts for more than 12 months,
- Reporting of all serious adverse events related to this study to the ERC within three days verbally and seven days in writing.
- Submission of a final report after completion of the study
- Informing ERC if study cannot be implemented or is discontinued and reasons why
- Informing the ERC and your sponsor (where applicable) before any publication of the research findings.

You are kindly advised to adhere to the national guidelines or protocols on the prevention of COVID -19

Please note that any modification of the study without ERC approval of the amendment is invalid.

The ERC may observe or cause to be observed procedures and records of the study during and after implementation.

Kindly quote the protocol identification number in all future correspondence in relation to this approved protocol

SIGNED................Bannerman..............................
Dr. Cynthia Bannerman
(GHS-ERC Chairperson)

Cc: The Director, Research & Development Division, Ghana Health Service, Accra
