# Supplementary material for: Integrating palliative care into primary care for older people with multimorbid serious illness: a multinational qualitative cross-sectional study in Sub-Saharan Africa
Source: BMJ Public Health. 2025 Mar 23;3(1):e001355. doi: 10.1136/bmjph-2024-001355 (PMC11934398; doi:10.1136/bmjph-2024-001355)
Supplement: online supplemental file 4 [file bmjph-3-1-s004.pdf]

Telephone: 08644072773/791193
E-mail: mrcz@mrcz.org.zw
Website: http://www.mrcz.org.zw

Medical Research Council of Zimbabwe
Josiah Tongogara / Mazowe Street
P. O. Box CY 573
Causeway

APPROVAL

MRCZ/A/2759 **27 July, 2021**

Dr Dickson Chifamba
Island Hospice and Healthcare
6 Natal Road, Belgravia
Harare

RE: MAP-Care: Multimorbid Ageing Primary Palliative Care in Ghana, Malawi and Zimbabwe

Thank you for the application for review of research activity that you submitted to the Medical Research Council of Zimbabwe (MRCZ). Please be advised that the Medical Research Council of Zimbabwe has **reviewed** and **approved** your application to conduct the above titled study.

This approval is based on the review and approval of the following documents that were submitted to MRCZ for review:

1. Full Protocol, Version 2, dated 8 July 2021
2. Interview Guide Caregivers Version Objective 5 and 6, (English, Shona, and Ndebele) Version 1 dated 23 May 2020
3. Interview Guide for Patient Caregivers Version Cognitive Interviews (Objective 4), (English, Shona, and Ndebele) Version 1 dated 23 May 2020
4. Patient – and Care Cancer Cost (PaCCCt) Survey, (English, Shona, and Ndebele) Version 1 dated 23 May 2020
5. Intervention Questionnaire, (English, Shona, and Ndebele) Version 1 dated 23 May 2020
6. Topic Guide for Objective II: Caregivers Version in Depth Interviews Phase 1, (English, Shona and Ndebele Version 2 dated 23 May 2020
7. Topic Guide for Objective II: In Depth Interviews for Health Professionals Phase 1, (English, Shona, and Ndebele) Version 1 dated 23 May 2020
8. Topic Guide For objective II: In Depth Interviews for Patients Phase 1, (English, Shona, and Ndebele) Version1 dated 23 May 2020
9. Interview Guide for Health Professionals Objective 5 and 6, (English, Shona and Ndebele Version 1 dated 23 May 2020
10. Information Sheet and Consent Form: Expert Group Meeting, (English, Shona, and Ndebele) Version 1 dated 23 May 2020
11. Information Sheet and Consent Form: Patient Participants (Intervention Feasibility), Phase 2 Objective (5 and 6), (English, Shona, and Ndebele) Versions 1 dated 23 May 2020)
12. Information Sheet and Consent Form: Healthcare Professionals Focus Group Discussion, Phase 2 Objective (5 and 6), (English, Shona, and Ndebele) Version 1 dated 23 May 2020)
13. Information Sheet and Consent Form: Patient in depth interviews, Phase 2 Objective (5 and 6), (English, Shona, and Ndebele) Version 1 dated 23 May 2020)
14. Information Sheet and Consent Form: Caregivers Versions/Family Members in depth interviews, Phase 2 Objective (5 and 6), (English, Shona, and Ndebele) Version 1 dated 23 May 2020)
15. Information Sheet and Consent Form: Patient Cognitive interviews, Phase 1 Objective 4, (English, Shona, and Ndebele) Version 1 dated 23 May 2020)
16. Information Sheet and Consent Form: Caregivers Versions/Family Members Cognitive interviews, Phase 1 Objective 4, (English, Shona, and Ndebele) Version 1 dated 23 May 2020)
17. Information Sheet and Consent Form: Healthcare Professionals – Focus Group interviews, Phase 1 Objective 2, (English, Shona, and Ndebele) Version 1 dated 23 May 2020)
18. Information Sheet and Consent Form: Caregivers Versions/Family Members in depth interviews, Phase 1 Objective 2, (English, Shona, and Ndebele) Version 1 dated 23 May 2020)
19. Information Sheet and Consent Form: Patient in depth interviews, Phase 1 Objective 2, (English, Shona, and Ndebele) Version 1 dated 23 May 2020)

- **APPROVAL NUMBER** : **MRCZ/A/2759**

This number should be used on all correspondence, consent forms and documents as appropriate.

- **TYPE OF MEETING** : **Expedited**
- **APPROVAL DATE** : **27 July, 2021**
- **EXPIRATION DATE** : **26 July, 2022**

After this date, this project may only commence upon renewal. For purposes of renewal, a progress report on a standard form obtainable from the MRCZ Offices should be submitted three months before the expiration date for continuing review.

- **SERIOUS ADVERSE EVENT REPORTING**: All serious problems having to do with subject safety must be reported to the Institutional Ethical Review Committee (IERC) as well as the MRCZ within 3 working days using standard forms obtainable from the MRCZ Offices or website.
- **MODIFICATIONS**: Prior MRCZ and IERC approval using standard forms obtainable from the MRCZ Offices is required before implementing any changes in the Protocol (including changes in the consent documents).
- **TERMINATION OF STUDY**: On termination of a study, a report has to be submitted to the MRCZ using standard forms obtainable from the MRCZ Offices or website.
- **QUESTIONS**: Please contact the MRCZ on Telephone No. (0242) 791193/08644073772 or by e-mail on mrcz@mrcz.org.zw

Other

- Please be reminded to send in copies of your research results for our records as well as for Health Research Database.
- You're also encouraged to submit electronic copies of your publications in peer-reviewed journals that may emanate from this study.
- In addition to this approval, all clinical trials involving drugs, devices and biologics (including other studies focusing on registered drugs) require approval of Medicines Control Authority of Zimbabwe (MCAZ) before commencement.

Yours Faithfully

SRugare

....

MRCZ SECRETARIAT
FOR CHAIRPERSON
MEDICAL RESEARCH COUNCIL OF ZIMBABWE

MEDICAL RESEARCH COUNCIL OF ZIMBABWE
2021 -07- 27
APPROVED
[illegible] CAUSEWAY, HARARE

PROMOTING THE ETHICAL CONDUCT OF HEALTH RESEARCH
