# Supplementary material for: Integrating palliative care into primary care for older people with multimorbid serious illness: a multinational qualitative cross-sectional study in Sub-Saharan Africa
Source: BMJ Public Health. 2025 Mar 23;3(1):e001355. doi: 10.1136/bmjph-2024-001355 (PMC11934398; doi:10.1136/bmjph-2024-001355)
Supplement: online supplemental file 5 [file bmjph-3-1-s005.pdf]

UNIVERSITY OF MALAWI

CERTIFICATE OF ETHICS APPROVAL

This is to certify that the College of Medicine Research and Ethics Committee (COMREC) has reviewed and approved a study entitled:

P.08/20/3108 - MAP-care: Multimorbid Ageing Primary Palliative Care in Ghana, Malawi and Zimbabwe. by Richard Harding

On 08-Oct-20

As you proceed with the implementation of your study, we would like you to adhere to international ethical guidelines, national guidelines and all requirements by COMREC some of which are indicated on the next page for your study

08-Oct-20

Prof. E. Umar -Chairperson (COMREC)

Date

Approved by
College of Medicine
08-Oct-2020
(COMREC)
Research and Ethics Committee
