# Supplementary material for: Integrating palliative care into primary care for older people with multimorbid serious illness: a multinational qualitative cross-sectional study in Sub-Saharan Africa
Source: BMJ Public Health. 2025 Mar 23;3(1):e001355. doi: 10.1136/bmjph-2024-001355 (PMC11934398; doi:10.1136/bmjph-2024-001355)
Supplement: online supplemental file 6 [file bmjph-3-1-s006.pdf]

Table 3: Demographic characteristics of the study participants n=135

**Older people n=41**	
Mean age (SD)	62.3 (10.5)
Sex **n (%)**	
Female	27 (65.9)
Male	14 (34.1)
Education (1 missing) **n (%)**	
No education	11 (27.5)
Primary School	11 (27.5)
Secondary school	13 (32.5)
Tertiary school	5 (12.5)
Religion **n (%)**	
Christianity	20 (48.8)
Islam	11 (26.8)
Orthodox	10 (24.4)
Occupation **n (%)**	
Not employed	24 (58.5)
Farmer	11 (26.8)
Business	4 (9.8)
Temporary employment	2 (4.9)
Diagnosis **n (%)**	
Hypertension and diabetes	11(26.8)
Stroke, hypertension and diabetes	8 (19.5)
HIV and cancer	6 (14.6)
Cancer, hypertension, arthritis	5 (12.2)
Hypertension, diabetes and cancer	4 (9.8)
Heart failure and hypertension	4 (9.8)
Cancer, hypertension, arthritis	3(7.3)
Country **n (%)**	
Ghana	12 (29.3)
Malawi	15 (36.6)
Zimbabwe	14 (34.1)

**Caregiver participants n=37**	
Mean age (SD)	40.8 (3.82)
Sex **n (%)**	
Female	29 (78.4)
Male	8 (21.6)
Relationship with patient **n (%)**	
Sibling	13 (35.1)
Parent	10 (27.1)
Partner/husband/wife	6 (16.2)
Son/daughter/niece/granddaughter	5 (13.5)
Grandparent	3 (8.1)
Education **n (%)**	
No education	2 (5.4)
Primary school	9 (24.3)
Secondary school	19 (51.4)
Tertiary school	7 (18.9)
Religion **n (%)**	
Christianity	24 (64.9)
Islam	9 (24.3)
Orthodox	4 (10.8)
Occupation **n (%)**	
Not employed	10 (27.1)
Farmer	9 (24.3)
Full-time employment	7 (18.9)
Student	5 (13.5)
Casual labour	3 (8.1)
Business	3 (8.1)
Country **n (%)**	
Ghana	10 (27.1)
Malawi	12 (32.4)
Zimbabwe	15 (40.5)
**Staff participants n=57**	
Age range	18-70

Sex **n (%)**	
Female	42 (73.7)
Male	15 (26.3)
Occupation **n (%)**	
Nurse	24 (42.1)
Community health workers	9 (15.8)
Pharmacy assistant	5 (8.8)
Clinical officers	4 (7)
Hospital attendant	4 (7)
Medical assistants	3 (5.3)
Environmental health officers	2 (3.5)
Nutritionists	2 (3.5)
Social worker/Clinical psychologists	2 (3.5)
Medical doctor	1 (1.75)
Physiotherapists	1 (1.75)
Mean (SD) years working at the facility	7.4 (5.3)
Mean (SD) years of work experience	10.1 (6.6)
Country **n (%)**	
Ghana	12 (21.1)
Malawi	22 (38.5)
Zimbabwe	23 (40.4)
