# Supplementary material for: Integrating palliative care into primary care for older people with multimorbid serious illness: a multinational qualitative cross-sectional study in Sub-Saharan Africa
Source: BMJ Public Health. 2025 Mar 23;3(1):e001355. doi: 10.1136/bmjph-2024-001355 (PMC11934398; doi:10.1136/bmjph-2024-001355)
Supplement: online supplemental file 7 [file bmjph-3-1-s007.pdf]

Table 4: Main themes, subthemes and Quotes

Theme & subtheme	Quote
1) Communication<br><br>1a) Decision making	Quote 1: "The patients makes the decision most of the time, but sometimes I disagree with them because he has been taking medicine for a long time and he sometimes gets confused but for us who are fine we can make better decisions and help him" (Caregiver, C004; Malawi)<br><br>Quote 2: I take a big part in decision making because he's my patient......for example last week we went to the hospital, it's me who made the decision.....he was refusing to go, he was saying that they will not attend to us because we go to the hospital frequently but I encouraged him (Caregiver, C005, Ghana)<br><br>Quote 3: When we visit the hospital some doctors ask us [during the consultation] like " can we do this and that?" . For me it feels good because I know that am included in the discussion. (Caregiver, C006, Zimbabwe)
1b) Communication styles	Quote 4: We see that they treat us properly because when we arrive, they greet us and take us to where they want us to be (Caregiver, C014, Malawi).<br>Quote 5: Even if she is busy, she would at least pause a little to listen and talk to you (Caregiver C011, Ghana)<br><br>Quote 6: Sometimes the patients they fail to voice out their concerns because the attitude that we health workers have. You find somebody welcomes the patient well and treats him or her holistically. That makes the patient to have the ability to explain what complaints one has. So patients also see differences in us (Healthcare professional, Malawi)
1c) Information provision	Quote 7: I asked him [the doctor]'You are telling us this is cancer, but your fellow doctors who have been examining my husband at the beginning of this illness did not tell us what you are telling us today, we have been meeting those doctors several times' (Caregiver C005, Malawi)<br><br>Quote 8: Yes, she explained, how the disease will affect me and how to use the colostomy bag and prevent possible complications. She told me to what extent the bag was allowed to be full or else it would burst and spill the contents.<br>(Patient 011, Zimbabwe)<br><br>Quote 9: I think the information that we tell them is just too much for someone to understand like in a short duration, they need to be told again and again like someone never told them before. Like we need to repeat the same things that were said even on the first visit (Health Professional, Zimbabwe).
1d) Communication barriers	Quote 10: No, I haven't reached that extent to ask, because the doctor is the one who tells me what he finds after examining me, then I don't ask anything, like "what about my lungs" no.[...] I just feel it will be like I am commanding them on their duty (Patient 008, Ghana)<br><br>Quote 11: Most patients have challenges in communicating. You will find, maybe of course there is a language barrier on one hand, and on the other hand is like maybe because they are chronically sick, some of them they are depressed with

	the illness, they are tired, they are having side effects, they are getting a lot of medication, they can't remember (Health professional, Ghana).
2) Coordination of care	Quote 12: There is unity of purpose among themselves so much so that if the one who attended to you in the first place is not around, they are able to discuss your care over the phone (Caregiver 001, Malawi).<br><br>Quote 13: Well, is well coordinated is like, although she sees a different person, every time is more coordinated, you could see the progression of care, except that you meet most often or sometimes you meet a different person, each time (Caregiver, 004, Ghana).<br><br>Quote 14: There is no clear coordination when it comes to receiving medication because we are assisted by different doctors when we visit the hospital which makes it difficult for the doctors to keep track of the medication. It would have been better for one doctor to keep track of medication of one patient (Patient, 005; Malawi).<br><br>Quote 15: When is time for us to go to the clinic, sometimes we leave here at 5am to 5:30 am. We will get there around 7am and have to wait before the nurses come. You will wait till 10am before the nurses come and check the blood pressure so you become tired. (Caregiver C001Ghana).<br><br>Quote 16: What I wanted to be treated urgently is the pains of the joints because this problem troubles me a lot. If it can resolve as quickly as it did last time, I would say soon I would be going back to my work of construction. (Patient 001, Malawi).
3) Impact of illness<br>3a) On activities of daily living	Quote 17: I face challenges if I am to travel, for any long journey I must punish myself by not eating, because if I eat a lot of stool comes out of the stoma! I was advised to drink plenty of fluids at least 2 litres per day so travelling becomes a struggle. If I am going where I am going to spend part of the day, most of the times I do not eat because I am afraid that my stoma bag start filling up very fast (Patient, 011, Zimbabwe)<br><br>Quote 18: I can say that our main priority is food for her, because it is not every time that we can manage to get the food she needs, because sometimes it happens that I don't have money but she wants something. Of course, sometimes we try to provide what she wants, but not all the time. then I feel that according to her life with the advice that she was given about the type of food which she should eat, sometimes we fail to follow because we don't have money to buy those things, then I feel that this is a big problem. (Caregiver, Malawi 006).
3b) Finances	Quote 19: The medical aid funds were exhausted so could not cover a $6000 shortfall (Caregiver C011, Zimbabwe)<br><br>Quote 20: Sometimes she sacrifices her medication paying money at the university. She has many issues which takes most of her money. At times there isn't enough money to buy her pills, so she ends up getting pills for diabetes only and not for hypertension. She just says they wasn't enough money to get both (Caregiver C005, Ghana)<br><br>Quote 21: I decided to come here in my village [rural area], because I want to get money for rentals from my house (Patient 007, Zimbabwe)

	Quote 22: If I have my stoma bags, I do not have any problem. These bags are my main problem. I stress a lot when I do not have them. I cannot travel without the bags! When I am going to church, I should have an extra bag inside my purse so, that if anything happens, I will go to the toilet to change. This is what troubles me, this is my main need (Patient, P011, Zimbabwe)
4) Seeking healthcare	Quote 23: We have a geriatric clinic here for old age from 65 years and above so I'm trying to bring her here to help (Caregiver, 001, Ghana)<br><br>Quote 24: Most of the times when you go to the outpatient's clinic nowadays you lined or booked according to the clinics you are attending and the days of the week (Patient, 010, Zimbabwe)<br><br>Quote 25: What I think is that when we go to the hospital/clinics, older patients should be prioritized like what they do in the Post office, they usually prioritize older people and those with ill health in case they collapse in the queues, so they are told to go to the front of the queue<br>(Patient 011, Zimbabwe)
5) Living with chronic illness, coping strategies and resources<br><br>5a) Accepting the illness and spiritual care	Quote 26: I just appreciate the treatment I am getting because when one lives the type of life I am living, relying on medication from 2015, at the end of the day you just accept it, so now I just know that I rely on my medication to live. My children were informed and accepted it, they know there are medication required and they are supposed to buy them.<br>(Patient 002, Zimbabwe)<br><br>Quote 27: About life I know that I am a chronically ill person. I have fully accepted my condition to the extent that I never show people that I am a sick person. I have embraced my illness.<br>My friends and family have accepted it. They know that I have cancer and I am a patient (Patient 011, Zimbabwe).<br><br>Quote 28:<br>Illness has brought me closer to my to God....daily prayers, hoping and praying that she will stay stronger, healthier each day, that we are gifted with (Caregiver 004, Ghana)
5b) Material and financial support	Quote 29: And some of them support her with what they have, especially things to do with food because she was restricted to eat certain foods and the relatives assist us with money to buy foods like milk (Caregiver 15, Malawi)<br><br>Quote 30: Because, as it stands now, it is my family and children that support me financially especially, so if there is any gift someone wants to give, I'm ready to accept it. But I'm looking forward to the continuous support from my children and not others (Patient 001, Ghana).
5c) Informal practical support and care	Quote 31: Yes, they help me a lot, they even cultivated my farm for me so I expect to harvest my own maize. They told me not to do any farming activities because that could worsen the wound in me (cervical cancer) (Patient 008, Zimbabwe)<br><br>Quote 32: They help me because they are my relatives whenever they get something (food, money) they give me, most of the time they give me cooked food to eat because I cannot manage to cook by myself, it is better these days am a

	little bit fine than those past days. Things were not good (was not fine). (Patient 010, Ghana)
